# Supplementary material for: Opioid Addiction and Opioid Receptor Dimerization: Structural Modeling of the OPRD1 and OPRM1 Heterodimer and Its Signaling Pathways
Source: Int J Mol Sci. 2021 Sep 24;22(19):10290. doi: 10.3390/ijms221910290 (PMC8509015; doi:10.3390/ijms221910290)
Supplement: Supplementary file 1 [file ijms-22-10290-s001.zip › Khalyfa-MCT2 supple- 08-14-2021.pptx]

## Slide 1
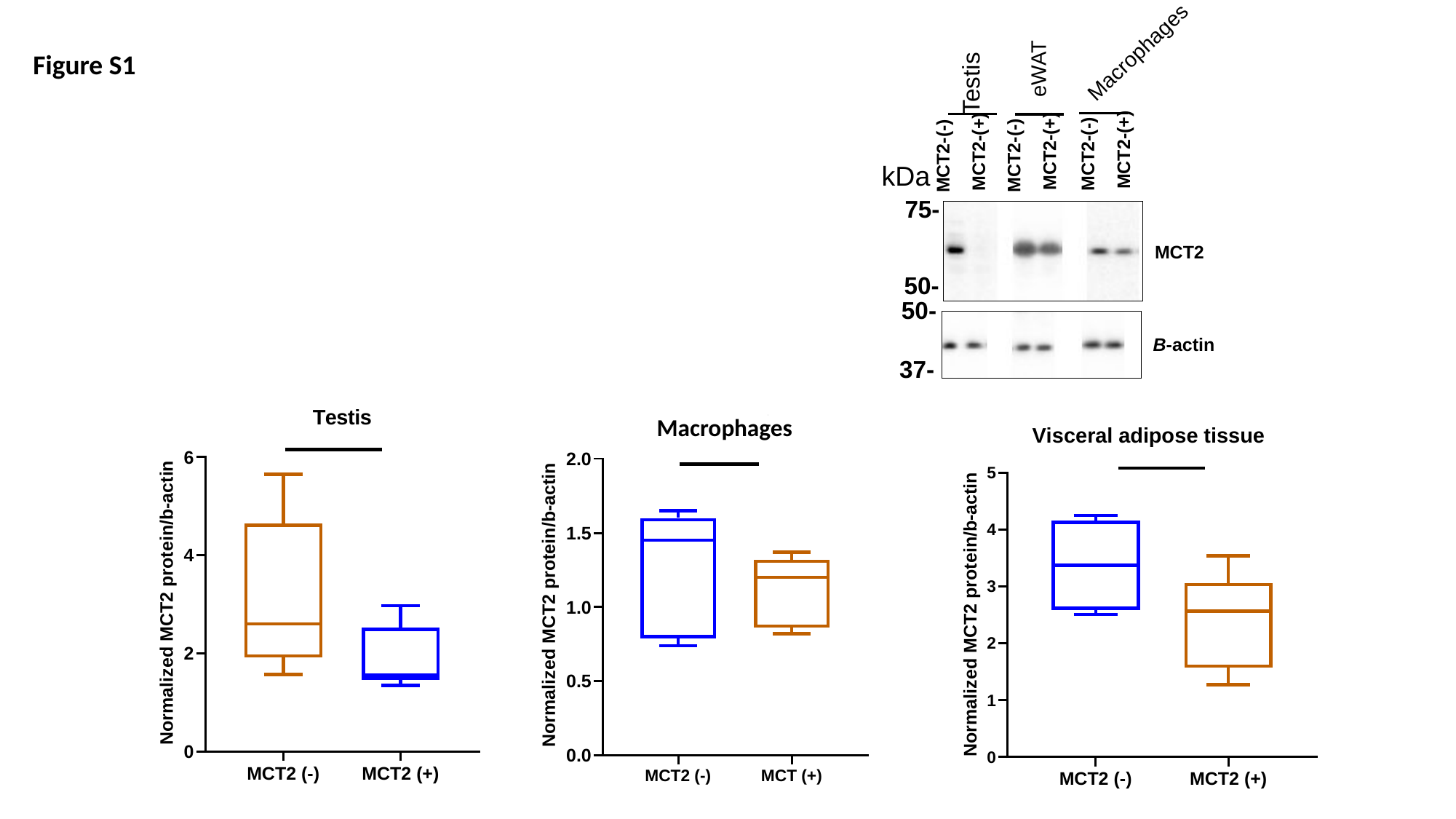

Macrophages
eWAT
Testis
MCT2-(+)
MCT2-(+)
MCT2-(+)
MCT2-(-)
MCT2-(-)
MCT2-(-)
75-
MCT2
50-
50-
B-actin
37-
Figure S1
Macrophages
kDa

## Slide 2
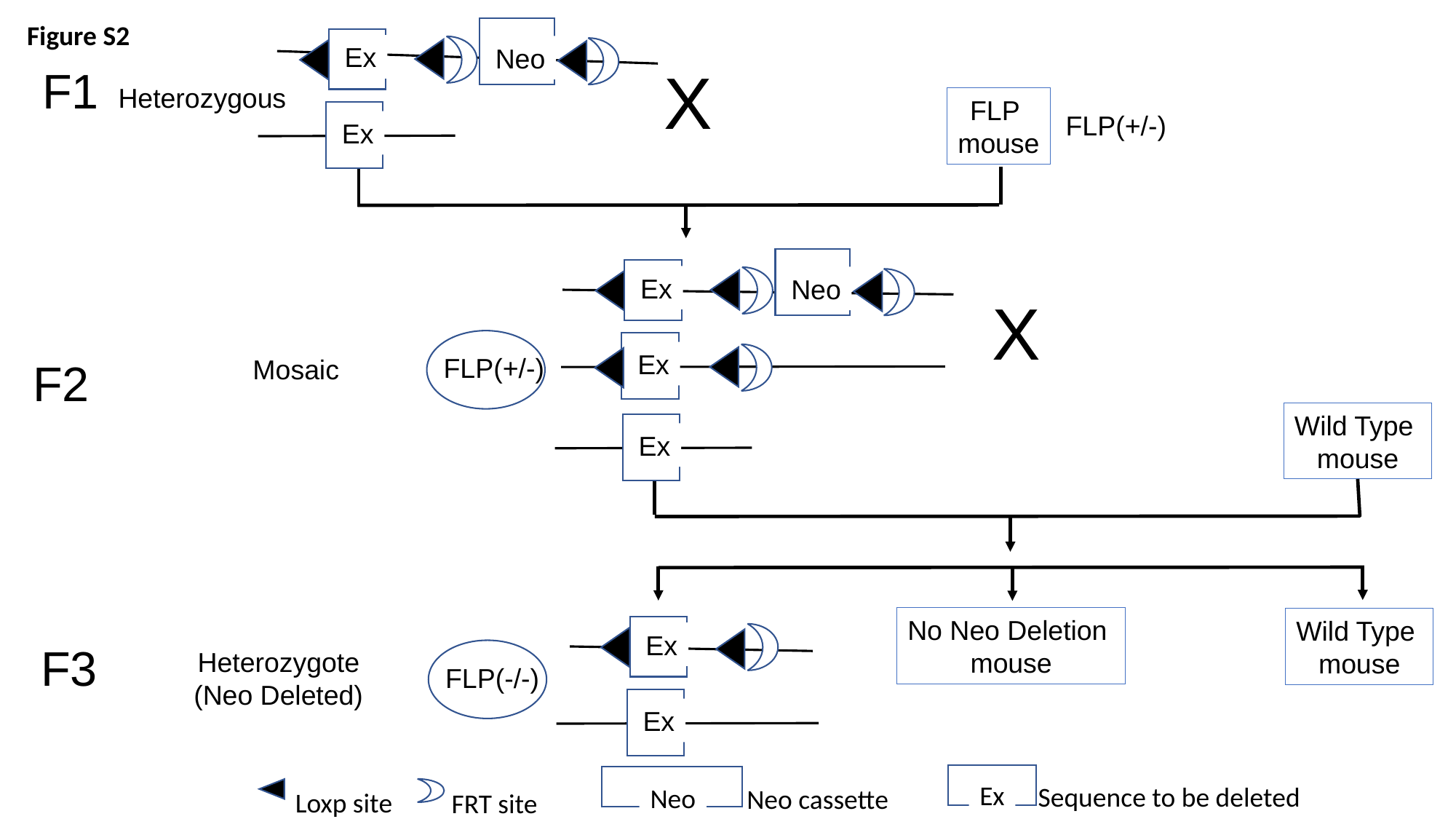

Figure S2
Ex
Neo
X
F1
Heterozygous
FLP
mouse
FLP(+/-)
Ex
Ex
Neo
X
Ex
FLP(+/-)
Mosaic
F2
Wild Type
mouse
Ex
No Neo Deletion
mouse
Wild Type
mouse
Ex
F3
Heterozygote
(Neo Deleted)
FLP(-/-)
Ex
Ex
Sequence to be deleted
Neo
Neo cassette
Loxp site
FRT site

## Slide 3
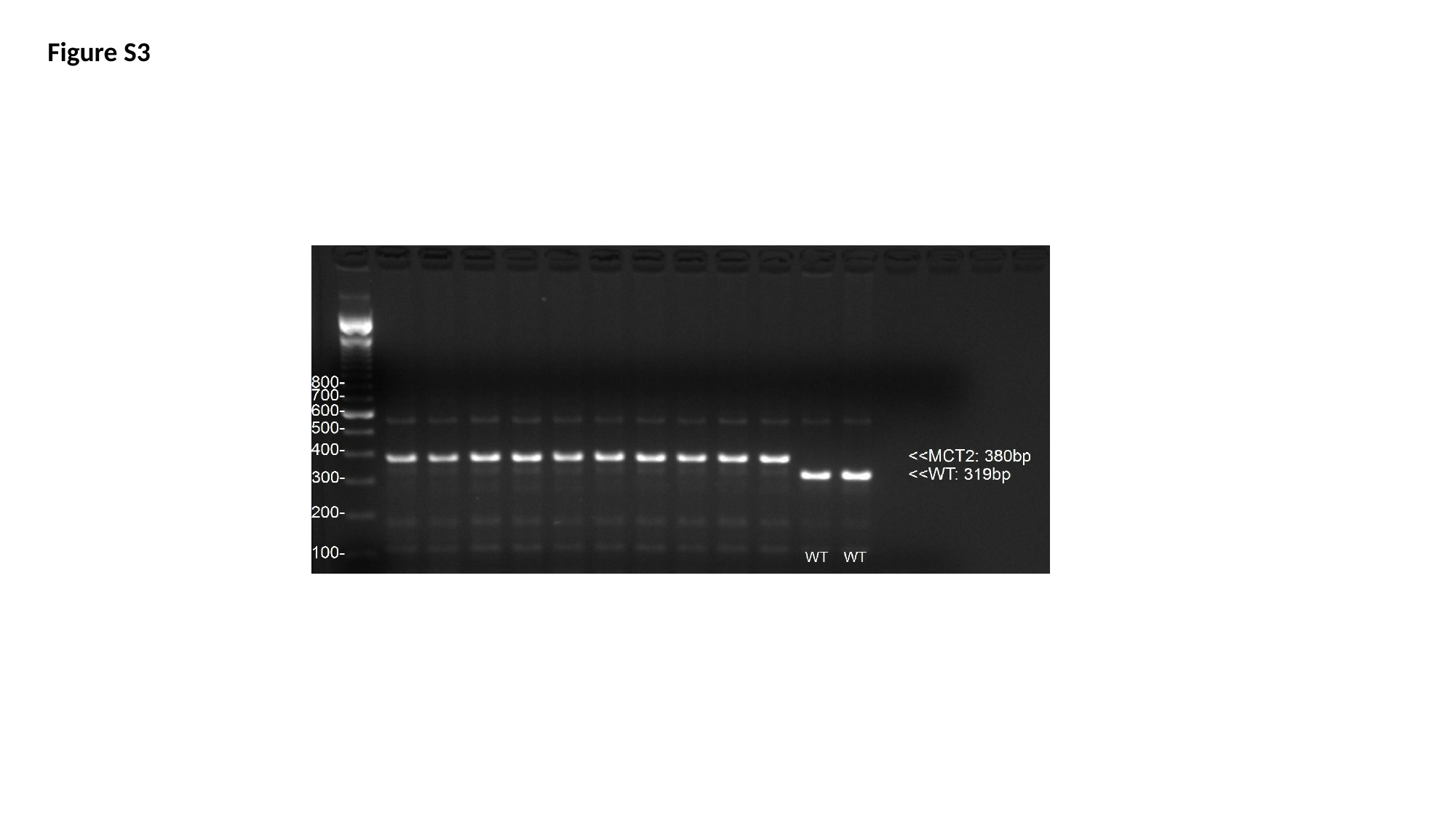

Figure S3
